# Effects of a Brown Beans Evening Meal on Metabolic Risk Markers and Appetite Regulating Hormones at a Subsequent Standardized Breakfast: A Randomized Cross-Over Study

**DOI:** 10.1371/journal.pone.0059985

**Published:** 2013-04-05

**Authors:** Anne Nilsson, Elin Johansson, Linda Ekström, Inger Björck

**Affiliations:** Division of Applied Nutrition and Food Chemistry, Department of Food Technology, Engineering and Nutrition, Lund University, Sweden; National Institute of Agronomic Research, France

## Abstract

**Background:**

Dietary prevention strategies are increasingly recognized as essential to combat the current epidemic of obesity and related metabolic disorders. The purpose of the present study was to evaluate the potential prebiotic effects of indigestible carbohydrates in Swedish brown beans (*Phaseolus vulgaris* var. *nanus)* in relation to cardiometabolic risk markers and appetite regulating hormones.

**Methods:**

Brown beans, or white wheat bread (WWB, reference product) were provided as evening meals to 16 healthy young adults in a randomised crossover design. Glucose, insulin, appetite regulatory hormones, GLP-1, GLP-2, appetite sensations, and markers of inflammation were measured at a following standardised breakfast, that is at 11 to 14 h post the evening meals. Additionally, colonic fermentation activity was estimated from measurement of plasma short chain fatty acids (SCFA, including also branched chain fatty acids) and breath hydrogen (H_2_) excretion.

**Results:**

An evening meal of brown beans, in comparison with WWB, lowered blood glucose (−15%, p<0.01)- and insulin (−16%, p<0.05) responses, increased satiety hormones (PYY 51%, p<0.001), suppressed hunger hormones (ghrelin −14%, *p*<0.05), and hunger sensations (−15%, *p* = 0.05), increased GLP-2 concentrations (8.4%, *p*<0.05) and suppressed inflammatory markers (IL-6 −35%, and IL-18 −8.3%, *p*<0.05) at a subsequent standardised breakfast. Breath H_2_ (141%, *p*<0.01), propionate (16%, *p*<0.05), and isobutyrate (18%, P<0.001) were significantly increased after brown beans compared to after WWB, indicating a higher colonic fermentative activity after brown beans.

**Conclusions:**

An evening meal with brown beans beneficially affected important measures of cardiometabolic risk and appetite regulatory hormones, within a time frame of 11–14 h, in comparison to a WWB evening meal. Concentrations of plasma SCFA and H_2_ were increased, indicating involvement of colonic fermentation. Indigestible colonic substrates from brown beans may provide a preventive tool in relation to obesity and the metabolic syndrome.

**Trial Registration:**

ClinicalTrials.gov NCT01706042

## Introduction

A diet rich in whole grain, dietary fibre (DF), anti-inflammatory components (e.g. polyphenols and omega-3 fatty acids), and low-GI foods, constitutes a promising preventive strategy against the increasing epidemic of obesity, the metabolic syndrome, and type 2 diabetes [1]–[5]. Certain cereal foods which are rich in indigestible carbohydrates (DF and resistant starch, RS), e.g. barley kernels based products, appears to facilitate glucose regulation in a semi-acute perspective, e.g. after 10–12 h (e.g. from an evening meal to a subsequent breakfast) [6]–[8]. Previously we reported that the benefits on blood glucose regulation were positively related to breath hydrogen excretion [9] and plasma levels of butyrate [10], indicating a role of colonic fermentation. An additional finding was that perceived satiety correlated with breath hydrogen excretion [9]. This indicates that colonic fermentation of DF and RS may affect key parameters involved in the regulation of glucose metabolism and satiety.

Epidemiological studies have linked bean consumption to lower risk of overweight and obesity. Among American adults, bean consumers displayed a 23% lower risk of obesity, and additionally displayed a lower systolic blood pressure [11]. Studies in Brazilian adults have further indicated that a habitual diet including beans was associated with lower risk of overweight and obesity in both men (−13% ) and women (−14%) [12]. In addition, intake of non-oil-seed pulses improved markers of long term glycaemic control [13], as judged from meta-analysis of medium to long-term studies, including both subjects with and without diabetes. However, whereas beans seem to prevent obesity, no acute benefits of pulses (navy beans) were seen on postprandial appetite or energy intake [14]. Thus, the inverse relations between pulse consumption and obesity seen in epidemiological studies must be explained by other factors. The underlying mechanisms are yet unknown; however in addition to their low GI, legumes are good sources of indigestible carbohydrates such as DF, oligosaccharides and RS.

The purpose of the present study was to evaluate the potential prebiotic effects of indigestible carbohydrates in Swedish brown beans (*Phaseolus vulgaris* var. *nanus)* in relation to cardiometabolic risk markers and appetite regulating hormones. For this purpose sixteen healthy young adults were provided a brown bean- or reference white wheat bread (WWB) evening meal, respectively, followed by a standardised breakfast, using a randomized cross over design. At fasting, and in the postprandial phase (0–180 min) after the standardised breakfast, blood glucose, serum insulin, markers in blood of inflammatory tonus (Interleukin 6 (IL-6), IL-18, adioponectin), appetite regulatory hormones (oxyntomodulin (OXM), ghrelin, PYY, glucagon-like peptide-1 (GLP-1)), GLP-2, free fatty acids (FFA) and subjective appetite sensations were measured. In parallel, measures of colonic fermentative activity were determined from analysis of plasma short chain fatty acids ((SCFA), including also branched chain fatty acids (BCFA)) and breath hydrogen excretion.

## Materials and Methods

**Figure 1:**
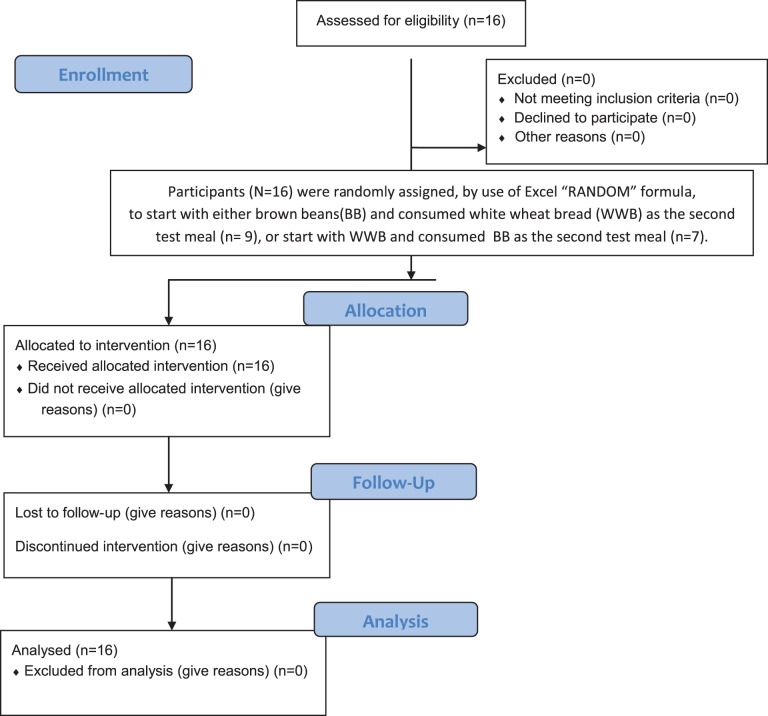
CONSORT flowchart, flow diagram of the study progress.

The trial protocol for this study and supporting CONSORT checklist are available as supporting information; see Protocol S1 and Checklist S1. The CONSORT flow diagram for the study is presented in **Figure 1**.

### Ethics Statement

Approval of the study was given by the Regional Ethical Review Board in Lund, Sweden (reference number 668/2008). Prior inclusion, written informed consent was obtained from each subject after a full explanation (written and oral) of the purpose and procedures of the study. All test subjects were aware of the possibility of withdrawing from the study at any time they desired.

### Test Subjects

Sixteen healthy volunteer subjects, 10 women and 6 men, with a mean age of 23.8±0.7 years (mean ± SEM) and mean BMI 22.5±0.6 kg/m^2^ (mean ± SEM) were recruited through local advertising at Lund University. Recruitments of test subjects were on going in February 2010, and the experimental work was finished in April 2010. All participants enrolled completed the whole study.

### The Evening Meal Test Product and the WWB Reference Bread

The test evening meal consisted of Swedish brown beans (Phaseolus vulgaris var. nanus), and a white wheat bread (WWB) was included as a reference evening meal. The serving sizes of beans and WWB were set in order to provide 35 g available starch. The amount of available starch was chosen to allow for a realistic meal size of beans. A pilot study indicated that a portion brown beans corresponding to 50 g available starch, commonly used for e.g. glycaemic index measurement, was not realistic for consumption as a late evening meal. However, due to the bulkiness of beans, glycaemic index measurements of bean products are performed at lower available carbohydrate levels e.g. 30 g [15]. The evening meals were prepared by the test subjects in their home according to a detailed written description of the cooking and thawing procedure of the beans and WWB, respectively. The brown beans were soaked in water for 12 hours before being boiled. The beans were boiled for 30 min, and consumed directly after preparation. The WWB was baked according to a standardized procedure in a home baking machine (Severin model nr. BM 3983; Menu choice, program 2 [white bread, 1000 g, quick (time2∶35)]). The bread was made from 540 g of white wheat flour (Kungsörnen Ab, Järna, Sweden), 360 g water, 4.8 g dry yeast, 4.8 g NaCl. After cooling, the crust was removed, the bread was sliced and portions (89 g) were wrapped in aluminium foil, put into plastic bags and stored in a freezer (−20°C). The test subjects received a serving of frozen bread to be put in their freezer without any delay. At the day of consumption of the WWB, the test persons were instructed to thaw the bread at ambient temperature, still wrapped in aluminium foil and in the plastic bag. Water, optional amounts, was consumed with the test- and reference products.

### Standardized Breakfast

A standardized breakfast was provided at the test days, consisted of a commercial white wheat bread corresponding to 50 g available carbohydrates with the crust removed (122 g Dollar storfranska, Lockarp, Sigvants bageri AB, Malmö, Sweden), and water (250 ml).

### Physiological Test Variables

Finger-prick capillary blood samples were taken for determination of blood glucose (HemoCue®B-glucose, HemoCue AB, Ängelholm, Sweden). Venous blood samples were collected to determine serum (s) insulin, s-FFA, s-IL-6, s-IL-18, s-adiponectin, plasma (p) GLP-1 (active 7–36), p-PYY, p-OXM, active p-ghrelin, p-GLP-2, and p-SCFA (inclusive BCFA).

Breath hydrogen was measured as an indicator of colonic fermentation, using an EC 60 gastrolyzer (Bedfont EC60 Gastrolyzer, Rochester, England). Pre- and post breakfast subjective appetite sensations were obtained using three scales for determination of subjective satiety, hunger, and the desire to eat, respectively. The subjects were supposed to mark on the scales, which were composed of 100 mm long lines where 0 mm denoted *not full at all, not hungry at all*, and *do not want to eat*, and 100 mm denoted *very full*, *extremely hungry,* and *very willing to eat*.

*Serum insulin* was determined with a solid phase two-site enzyme immunoassay kit (Insulin ELISA 10-1113-01, Mercodia AB, Uppsala, Sweden, *s-FFA* concentrations with an enzymatic colorimetric method (NEFA C, ACS-ACOD method, WAKO Chemicals GMbH, Germany). The quantitative determination of *s-IL-6* was performed with an enzyme immunoassay (Human IL-6 HS600B, R&D Systems, Abingdon, UK, and *IL-18* in serum with an enzyme immunoassay that was modified in the respect that no dilution of serum was performed prior to the analysis (Human IL-18 ELISA Kit 7620, MBL Medical & Biological Laboratories CO., Ltd, Nagoya, Japan). *Plasma adiponectin* concentrations were determined with a solid phase two-site enzyme immunoassay kit (Adiponectin ELISA 10-1193-01, Mercodia AB, Uppsala, Sweden), and the quantitative determination of bioactive *GLP-1* levels in plasma with a highly sensitive ELISA enzyme-linked immunosorbent assay kit (GLP-1 (Active 7–36) ELISA 43-GP1HU-EO1 ALPCO Diagnostics, Salem, NH). *Plasma GLP-2* concentrations were determined with a competitive enzyme immunoassay (Human GLP-2 EIA YK141, Yanaihara Institute Inc. Shizuoka, Japan), *p-ghrelin* with an enzyme immunometric assay (Human Acylated Ghrelin ELISA RD194062400R, BioVender GmbH, Heidelberg, Germany), and *p-OXM* with an immunoassay (Human Oxyntomodulin ELISA Kit XSB-E12948h, Cusabio Biotech CO., Ltd, Newark, USA). The concentrations of *PYY,* both PYY (3–36) and PYY (1–36) in plasma were determined with a competitive enzyme immunoassay (Human PYY EIA YKO8O, Yanaihara Institute Inc. Shizuoka, Japan). Plasma SCFA were analyzed using a GC method [16].

### Chemical Analyses of Test- and Reference Products

The test products were analyzed with respect to total starch [17], resistant starch (RS) [18], soluble- and insoluble dietary fibre [19], and raffinose (Kit Raffinose/Galactose K-RAFGA 07/11, Megazyme International Ireland Ltd) (**Table 1** and **Table 2**). The kit used was for quantification of Raffinose, however as well as raffinose, α-galactosidase in the kit also hydrolyses other α-galactosides, such as stachyose and verbascose. Before analysis of total starch and DF, boiled beans were air dried and milled (Cyclotec, Foss Tecator AB, Höganäs, Sweden). RS in the test products were analyzed on products as eaten. The available starch content was calculated by subtracting RS from total starch.

**Table 1:** Contents of starch (total and available) and dietary fibre in brown beans and WWB.

	Starch^1^		Dietary fiber^1^				
Products	Total	Available	Soluble	Insoluble	RS	Raffinose^2^	TotalDF^3^
––––––––––––––*% dry matter–––*––––––––––––							
Brownbeans	49	41	9.4	15	7.6	3.2	35
WWB	80	77	0.6	3.8	2.5	0.1	7.0

The values are presented as % dry matter.

1 Values of total starch are means of two replications, RS means of 6 replications, DF means of 3 replications.

2 The α-galactosidase in the kit used for quantification of raffinose, also hydrolyses other α-galactosides, such as stachyose and verbascose.

3 Included in total DF are insoluble and soluble dietary fibres determined by Asp et. al. (1983) [19], RS determined by the method described by Åkerberg et. al. (1998) [44], and Raffinose [45] as described above (section “Chemical analyses of test- and reference products”).

**Table 2:** Contents of starch (total and available) and dietary fibre in brown beans and WWB. The values are presented as gram per evening meal.

		Starch^1^		Dietary fiber^1^				
Evening meal	Portion size^2^	Total	Available	Soluble	Insoluble	RS	Raffinose^3^	Total^4^ DF
–––––––––––––––––*g/portion––*––––––––––––––––––-								
Brown beans	101	41	35	8.0	13.5	6.5	3.0	31
WWB	89	36	35	0.26	1.8	1.2	0.1	3.4

1 Values of total starch are means of two replications, RS means of 6 replications, DF means of 3 replications.

2 Weights of one test portion of uncooked beans and one portion of fresh bread.

3 The α-galactosidase in the kit used for quantification of raffinose, also hydrolyses other α-galactosides, such as stachyose and verbascose.

4 Included in total DF are insoluble and soluble dietary fibres determined by Asp et. al. (1983) [19], RS determined by the method described by Åkerberg et. al. (1998) [44], and Raffinose [45] as described above (section “Chemical analyses of test- and reference products”).

### Experimental Procedure

The study was designed as a randomized, crossover trial of the effect of brown beans consumed in the evening on cardiometabolic risk markers and appetite regulation at a following breakfast. A flow diagram of the study progress is presented in **Figure 1**. Participants were randomly assigned, by use of Excel “Random” formula, to start with beans or WWB. The subjects were encouraged to standardize their meal pattern, and to avoid food rich in DF the day before an experimental day. Furthermore, they should avoid alcohol and excessive physical exercise in the evening, and they should not have consumed antibiotics or probiotics for two weeks (no participants had consumed antibiotics during the last months). The test evening- and reference meals were consumed in a random order, separated by approximately one week. The experimental meals were consumed at 9 pm, and thereafter the subjects were fasting until the standardized breakfast. The subjects arrived at the experimental department (Applied Nutrition and Food Chemistry, Lund University) at approximately 07.45 the next morning, and an intravenous cannula (BD Venflon, Becton Dickinson, Helsingborg, Sweden) was inserted into an antecubital vein to be used for blood sampling. After 10 min resting in a sitting position, fasting blood tests were collected and subjective appetite sensations and breath H_2_ registered, and thereafter the standardized breakfast was served. The breakfast was consumed within 10 to 12 minutes. Physiological test markers were measured repeatedly during 3 h. Blood glucose and appetite sensations were determined fasting and every 15 min during the first hour, and then every half hour until 180 min after start of the breakfast. Insulin was determined fasting and every half hour until 120 min. Breath hydrogen was determined at every half hour until 180 min post breakfast. Plasma ghrelin, p-PYY, p-OXM, and p-GLP-2 were determined at fasting and at 30, 60, 120, and 180 min, and p-Glp-1 was determined at fasting and at 30, 45, 60, 120 and 180 min. Serum IL-6, s-IL-18, and s-adiponectine were determined at fasting and at 60, 120, and 180 min. Serum FFA was determined at fasting and at 180 min, and SCFA at fasting and at 60 min. The subjects were told to maintain a low physical activity throughout the 3 hours of blood sampling.

### Calculations and Statistical Methods

Data are expressed as means ± SEM. Incremental areas under the response curves (IAUC) for glucose and insulin, and areas under the curves (AUC) for GLP-1, ghrelin, and appetite sensations, were calculated for each subject and test meal, using the trapezoid model. GraphPad Prism (version 4 and 5, GraphPad Software, San Diego, CA, USA) was used for graph plotting and calculation of IAUC and AUC. The individual highest peak increase (Ipeak) in postprandial blood glucose concentration after the standardized breakfast was determined following each test meal, and the mean (n = 16) was used in comparison of effects of evening meals on peak glucose responses. Significant differences in test variables after the different test meals were assessed with ANOVA (general linear model), in MINITAB Statistical Software (release 14; Minitab, Minitab Inc, State College, PA). In the cases of non-normal distributed data (tested with Anderson-Darling and considered unevenly distributed when *P*<0.05), Box Cox transformation were performed on the data prior to the ANOVA. Differences between the products at different time points were evaluated using a mixed model (PROC MIXED in SAS release 9.2; SAS Institute Inc, Cary, NC) with repeated measures and an autoregressive covariance structure. Correlation analysis was conducted to evaluate the relation among dependent measures with the use of Pearson correlation in MINITAB Statistical Software (release 14; Minitab, Minitab Inc, State College, PA). For test variables where the variation in the concentration scarcely changed over time, a weighted mean were produced with means for each 60 min intervals. *P*-values ≤0.05 were considered statistically significant, n = 16.

### Power Calculation

Primary outcome measure was change in blood glucose incremental area under the curve 0–120 min (IAUC) after the standardised breakfast. Number of participants required for the study was determined in MINITAB, using previous results of “over-night” effects on glucose incremental area under the curve 0–120 min) (IAUC) of consuming barley kernel [9]. Assuming a difference of 70 mmol*min/L between after consuming WWB or brown beans the previous evening, and a SD of 82 mmol*min/L, with α = 0.05 and 1−β = 0.8, a number of 13–17 participants were required (two-tailed test). Therefore we chose to include 16 subjects.

## Results

### Blood Glucose and Serum Insulin

**Figure 2:**
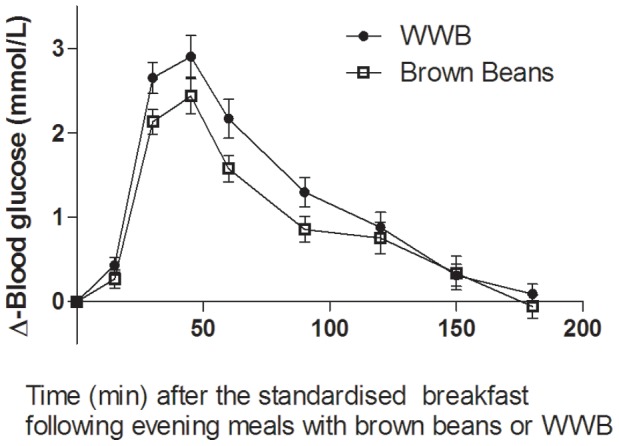
Mean incremental changes (Δ) in blood glucose concentrations after the standardized breakfast consumed 11 hours after evening meals composed of brown beans or WWB. A significant treatment (type of evening meal) effect was found over the test period (*p*<0.05, PROC MIXED in SAS release 9.2; SAS Institute Inc, Cary, NC). n = 16 subjects.

**Figure 3:**
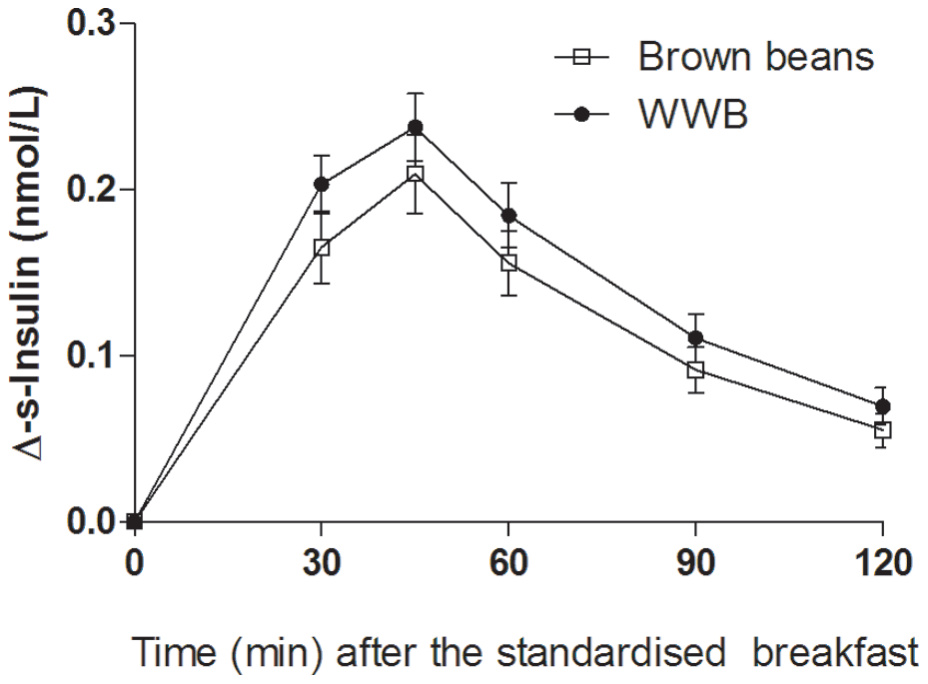
Mean incremental changes (Δ) in serum insulin concentrations after the standardized breakfast consumed 11 hours after evening meals composed of brown beans or WWB. A significant treatment (type of evening meal) effect was found over the test period (*p*<0.05, PROC MIXED in SAS release 9.2; SAS Institute Inc, Cary, NC). n = 16 subjects.

There were significant main effects of evening meals on glucose- and insulin responses (0–120 min, both: *p*<0.05) to the standardised breakfast (**Figure 2** and **Figure 3**). Glucose- and insulin IAUC (0–120 min) after the breakfast were decreased by 23% and 16%, respectively, after consumption of brown beans the previous evening, in comparisons with after the WWB evening meal (*p*<0.01 and *p*<0.05, respectively, **Table 3**). In addition, the glucose Ipeak responses to the standardised breakfast was lower (−15%) after the brown beans evening meal (*p*<0.05). The fasting glucose- and insulin concentrations were not affected by the different evening meals (*p* = 0.86, and *p* = 0.78, respectively).

**Table 3:** Results of the physiological test variables determined in the morning (fasting and/or postprandial a standardized breakfast) after consuming brown beans or WWB the previous evening.

Test variables	WWB	Brown Beans	%Δ^1^
Fasting blood glucose (mmol/L)	5.2±0.082	5.2±0.18	0
Blood Glucose IAUC (0–120 min, mmol*min/L)	192±14.5	147±8.74	−23*
Peak blood glucose elevation (mmol/L)	3.1±0.21	2.7±0.17	−15*
Fasting s-insulin (nmol/L)	0.039±0.003	0.038±0.004	−2.6
s-Insulin IAUC (0–120 min, nmol*min/L)	16.6±1.33	13.9±1.45	−16*
s-IL-6 (mean 0–180 min, ng/L)	1.37±0.202	0.89±0.062	−35*
s-IL-18 (mean 0–180 min, ng/L)	133±14.1	122±11.9	−8.3*
s-Adiponectine (mean 0–180 min, mg/L)	5.5±0.64	5.4±0.62	−2.5
p-PYY (mean 0–180 min, µg/L)	0.53±0.08	0.81±0.15	51*
p-OXM (mean 0–180 min, µg/L)	4.1±0.92	4.5±0.99	9.4†
p-Ghrelin AUC (0–180 min, µg*min/L)	7.6±0.8	6.6±7.5	−14*
p-GLP-1 AUC (0–180 min, pmol*min/L)	187±25.7	190±27.4	1.6
s-FFA (mean 0+180 min, mmol/L)	0.24±0.024	0.25±0.024	3.8
p-GLP-2 (mean 0–180 min, µg/L)	8.7±1.4	9.2±1.4	5.8
p-GLP-2 (mean 60–180 min, µg/L)	8.6±1.4	9.3±1.5	8.4*
p-GLP-2 (60 min, µg/L)	8.5±1.3	9.5±1.5	11*
Hunger AUC (0–45 min, m*min)	1.78±0.19	1.52±0.19	−15*
Satiety AUC (0–45 min, m*min)	2.19±0.20	2.43±0.23	11
Desire to eat AUC (0–45 min, m*min)	2.10±0.23	1.83±0.19	−13
p-Acetate (mean 0+60 min, µmol/L)	207±12	228±17	10
p-Propionate (mean 0+60 min, µmol/L)	11.8±0.66	13.7±0.84	16*
p-Isobutyrate (mean 0+60 min, µmol/L)	14.8±0.93	17.4±0.86	18*
p-Butyrate (mean 0+60 min, µmol/L)	11.8±0.39	12.7±0.60	7.6
p-Butyrate (0 min, µmol/L)	11.5±0.51	13.4±0.81	17†
p-Butyrate (60 min, µmol/L)	12.2±0.40	12.0±0.55	−1.6
Breath H2 (mean 0–180 min, ppm)	7.9±3	19±5	140*

1 % differences in concentrations of test marker in the mornings after brown beans evening meal compared with WWB evening meal.

* : *p*<0.05 (ANOVA) with respect to differences between results after brown beans compared to WWB,

† : *p* = 0.096 (ANOVA) with respect to differences between results after brown beans compared to WWB. p: plasma, s: serum.

### Inflammatory Markers in Serum (IL-6, IL-18, and Adiponectin) and s-FFA

**Figure 4:**
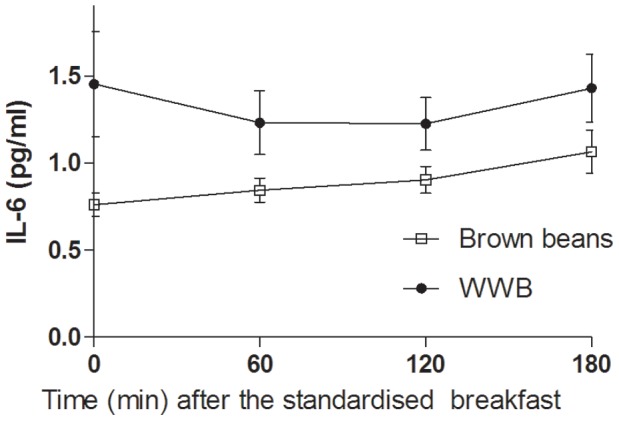
Concentrations of serum IL-6 fasting and after a standardized breakfast, consumed 11 hours after evening meals composed of brown beans or WWB. A significant treatment effect (type of evening meal) was found over the test period (*p*<0.05, PROC MIXED in SAS release 9.2; SAS Institute Inc, Cary, NC). n = 16 subjects.

**Figure 5:**
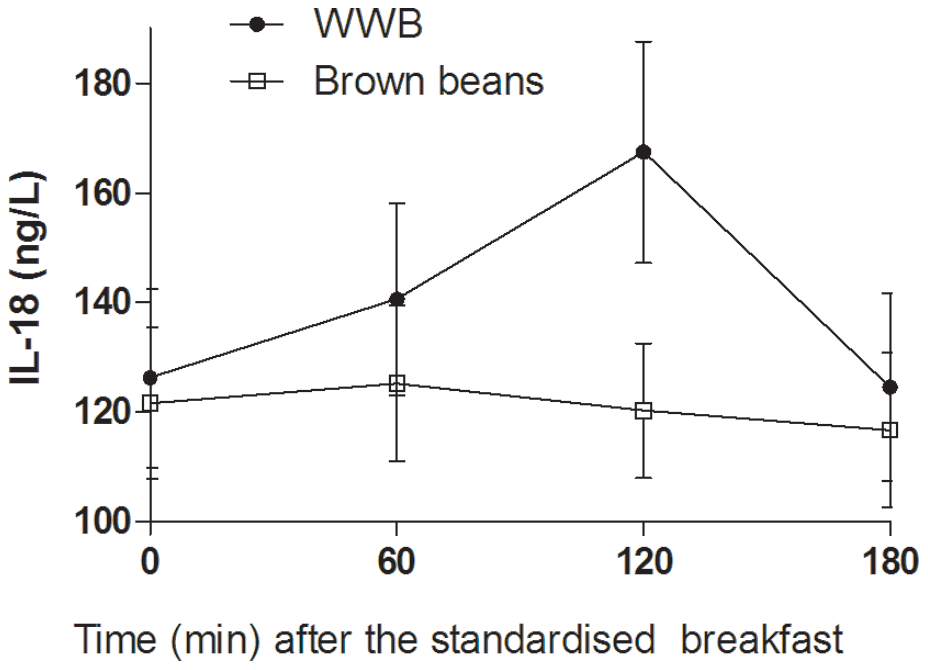
Concentrations of serum IL-18 fasting and after a standardized breakfast, consumed 11 hours after evening meals composed of brown beans or WWB. A significant treatment effect (type of evening meal) and treatment*time interaction were found over the test period (*p*<0.05 and *p*<0.001, respectively, PROC MIXED in SAS release 9.2; SAS Institute Inc, Cary, NC). n = 16 subjects.

Significant main effects of evening meals were seen in concentrations of inflammatory markers 0–180 min following the standardised breakfast (IL-6 and IL-18, *p*<0.05). The mean serum IL-6 concentration (0–180 min) after the breakfast was significantly lower following consumption of brown beans the previous evening compared to WWB **(** *p*<0.05, Table 3, **Figure 4**). A similar reduction was seen in the morning in IL-18 concentrations (mean 0–180 min) after the standardized breakfast following the brown beans evening meal (*p* = 0.05, Table 3, **Figure 5** **)**. In addition there was a meal*time interaction (*p*<0.001) in IL-18 concentrations, revealing most pronounced differences depending on evening meals at 120 min post breakfast (120 nmol/L and 167 nmol/L for brown beans and WWB, respectively, −28%, *p*<0.001).

No significant effects depending on evening meals were seen in adiponectin or FFA in the morning at fasting or following the standaridised breakfast (Table 3).

### Appetite Regulatory Hormones (p-PYY, p-OXM, p-ghrelin, p-GLP-1, and p-GLP-2)

**Figure 6:**
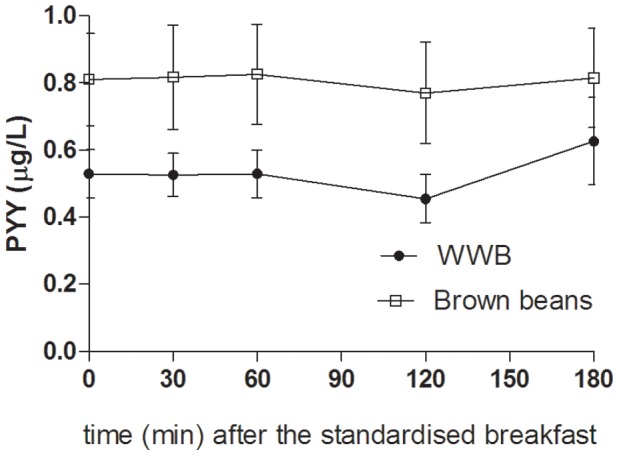
Concentrations of plasma PYY fasting and after a standardized breakfast, consumed 11 hours after evening meals composed of brown beans or WWB. A significant treatment effect (type of evening meal) was found over the test period (*p*<0.0001, PROC MIXED in SAS release 9.2; SAS Institute Inc, Cary, NC). n = 16 subjects.

**Figure 7:**
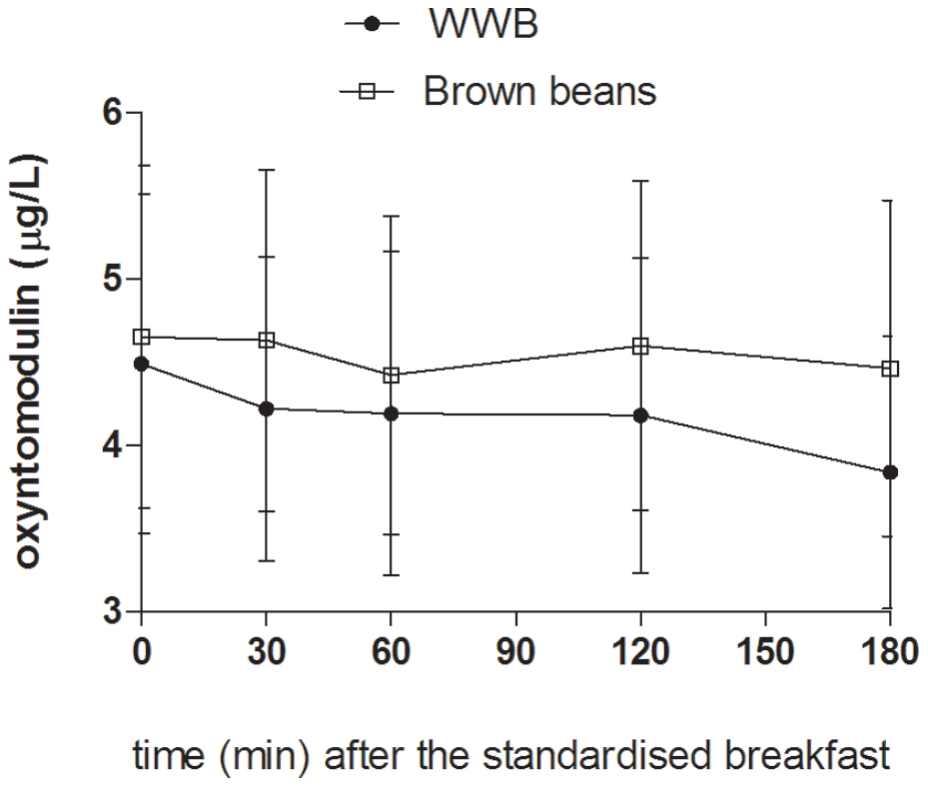
Concentrations of plasma oxyntomodulin fasting and after a standardized breakfast, consumed 11 hours after evening meals composed of brown beans or WWB. A significant treatment effect (type of evening meal) was found over the test period (*p*<0.05, PROC MIXED in SAS release 9.2; SAS Institute Inc, Cary, NC). n = 16 subjects.

The concentrations of appetite regulatory hormones in the morning were significantly affected by type of evening meal (main effects: PYY: *p*<0.0001, OXM: *p*<0.05, ghrelin: *p*<0.05, (**Figures 6** **,** **7** **, and** **8**). The brown bean evening meal resulted in 51% higher concentrations of PYY at fasting and following the standardised breakfast (mean 0–180 min: *p*<0.001, Table 3). Over the same time period, the mean concentrations of OXM tended to be higher after the brown beans (*p* = 0.086). Whereas an increase was seen in concentrations of anorexigenic peptides, the opposite was found with respect to the orexigenic peptide ghrelin. The concentrations of ghrelin (AUC 0–180 min post breakfast) in the morning following brown bean evening meal was significantly suppressed (−14%) in comparison with after the WWB evening meal (*p*<0.05, Table 3, Figure 7). No effects depending on evening meals were detected in fasting- or postprandial GLP-1 concentrations the following morning (Table 3).

**Figure 8:**
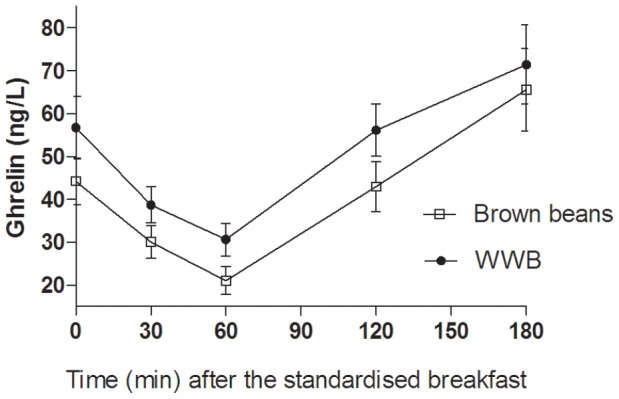
Concentrations of plasma ghrelin fasting and after a standardized breakfast, consumed 11 hours after evening meals composed of brown beans or WWB. A significant treatment effect (type of evening meal) was found over the test period (*p*<0.05, PROC MIXED in SAS release 9.2; SAS Institute Inc, Cary, NC). n = 16 subjects.

**Figure 9:**
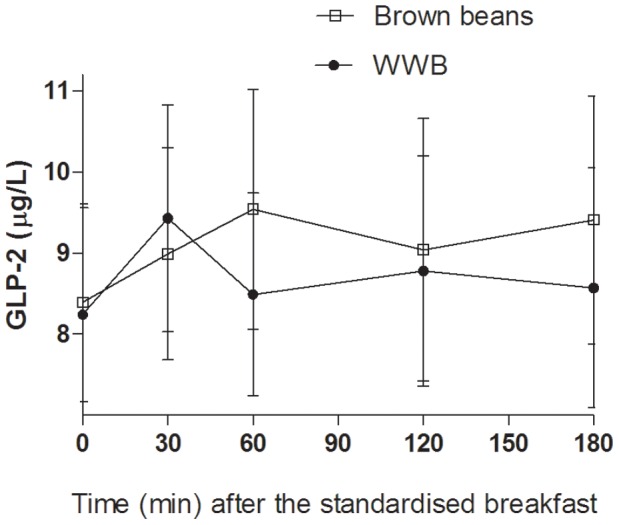
Concentrations of plasma GLP-2 fasting and after a standardized breakfast, consumed 11 hours after evening meals composed of brown beans or WWB. A tendency was found towards a meal*time interaction in the postprandial period after the standardized breakfast (*p* = 0.065, PROC MIXED in SAS release 9.2; SAS Institute Inc, Cary, NC), revealing a higher plasma GLP-2 in the later postprandial period when the subjects consumed brown beans as an evening meal compared with the WWB (60–180 min, *p*<0.05). n = 16 subjects.

No main effects depending on evening meals were seen in GLP-2 concentrations the following morning. However, there was a tendency towards a significant meal*time interaction in the postprandial period after the standardized breakfast (*p* = 0.065, **Figure 9**) making it relevant to investigate differences at different time points and intervals. Examination of the postprandial pattern of GLP-2 (0–180 min, Figure 9), revealed that the differences in GLP-2 depending on the evening meal, tended to increase in the later postprandial period after the standardised breakfast. Consequently, GLP-2 concentrations were significantly higher in the later postprandial period (60–180 min) when the subjects consumed brown beans as an evening meal compared with the WWB (9.3±1.5 and 8.6±1.4 µg/L for brown beans and WWB, respectively, *p*<0.05), whereas no significant differences were seen depending on evening meal in the early postprandial period (0–30 min: 8.7±1.3 and 8.8±1.4 µg/L for brown beans and WWB, respectively, *p* = 0.82). The differences in GLP-2 after brown beans and WWB were most pronounced at 60 min post the standardized breakfast (9.54±1.48 and 8.48±1.25 µg/L, respectively, table 3).

### Subjective Rating of Satiety, Hunger and Desire to Eat

No significant main effects of evening meals were detected on rating of appetite, hunger and willingness to eat when analysing results for the postprandial period 0–180 min post the standardised breakfast. However, in the early postprandial period, 0–45 min post breakfast, the feeling of hunger was rated lower when preceded by the brown bean evening meal as opposed to the WWB evening meal (AUC: 1.5±0.19 m*min and 1.8±0.19 m*min, respectively, *p* = 0.05, Table 3).

### Relationships between Appetite Regulating Hormones and Appetite Sensations

Relationships between satiety hormones and appetite sensations in the morning became most significant after consuming brown beans in the evening. Consequently, the willingness to eat (0–45 min) during the breakfast was inversely related to the plasma concentrations of PYY (0–180 min) (r = −0.50, *p*<0.05) and GLP-2 (r = −0.51, *p*<0.05). There was also a tendency towards a relation between plasma OXM and feeling of satiety (r = 0.48, *p* = 0.061).

### Plasma SCFA

The type of evening meal significantly affected concentrations of propionate and isobutyrate in plasma the following morning (main effects *p*<0.05 and *p*<0.001, respectively). ANOVA analysis showed that consumption of brown beans in the evening increased the concentrations of propionate and isobutyrate in comparison with an evening meal with WWB (mean 0+60 min: *p*<0.05 and *p*<0.001, respectively, table 3). There were no main effects of evening meal on concentrations of butyrate in the morning, but a significant treatment*time interaction (*p*<0.05) was detected, revealing a tendency (*p* = 0.096) towards higher butyrate concentrations at fasting following an evening meal with brown beans (Table 3). There were significant time effects in SCFA concentrations showing higher concentrations of isobutyrate at fasting (time = 0) compared with 60 min post breakfast (17.4±0.81 mmol/L and 14.7±0.62 mmol/L, respectively, *p*<0.001), meanwhile concentrations of propionate were higher at 60 min compared with at fasting (14.7±0.79 mmol/L and 12.6±0.98 mmol/L, respectively, *p*<0.001).

### Breath H_2_ Excretion

There was a significant main effect of evening meals on breath H_2_ excretion in the morning (*p*<0.0001). The mean breath H_2_ excretion (0–180 min) was significantly higher in the morning when brown beans have been consumed in the evening compared with the WWB (*p*<0.01, Table 3). After both evening meals, correlations were detected between H_2_ and SCFA. After WWB, breath H_2_ correlated with propionate (0+60 min) (H_2_ at fasting and at 0–180 min: r = 0.54, *p*<0.05) and with isobutyrate (0+60 min) (H_2_ at fasting: r = 0.54, *p*<0.05). In the morning after consuming brown beans the previous evening, breath H_2_ (0–180 min) correlated with plasma level of isobutyrate (0+60 min) (r = 0.54, *p*<0.05).

## Discussion

The main findings of this study were that an evening meal consisting of brown beans, in comparison with a WWB evening meal, lowered blood glucose- and insulin responses, increased satiety hormones (PYY, and trend for OXM), suppressed hunger hormones (ghrelin), and suppressed inflammatory markers (IL-6 and IL-18) at a proceeding standardised breakfast. Also plasma GLP-2 levels were increased at breakfast when preceded by the brown beans evening meal. The over-night stimulation on appetite regulatory hormones following brown beans was accompanied by a decreased sensation of hunger. Breath H_2_ concentrations, propionate, butyrate, and isobutyrate were significantly increased (trend for butyrate) after brown beans compared to after WWB, indicating a higher colonic fermentative activity after brown beans. An increase in formation of colonic SCFA is in accordance with previous studies in vitro [20] and in rat models [21], showing an increase in acetate, propionate and butyrate after common pulses. BCFA, such as isobutyrate, are more likely to origin from indigestible proteins [22], [23]. However, increased isobutyrate production has been demonstrated also after ingestion of certain DF (polydextrose) in human subjects [24].

Previously we have demonstrated similar beneficial over-night effects on cardiometabolic risk variables, e.g. on glucose tolerance [8], [9] and inflammatory markers [9], after consuming barley kernels based evening meals. However, the over-night suppressive effects on inflammatory markers, e.g. IL-6, was more evident with brown beans in comparison to barley kernels (−35% versus −17%, respectively). In contrast, in the current study no significant effects of a brown beans evening meal was seen in s-FFA or adiponectin, as previously seen with barley kernels.

The beneficial effects on cardiometabolic risk variables previously seen at breakfast, following barley kernels evening meals, were proposed to originate from events related to colonic fermentation of indigestible carbohydrates [9]. Also brown beans are a rich source of indigestible and potentially prebiotic carbohydrates. In accordance with the increased breath H_2_- and SCFA concentrations it is likely that the beneficial effects seen after brown beans stem from gut microbial fermentation. The brown bean evening meal in the present study corresponded to a total load of 31 g DF, including RS and oligosaccharides, of which a high proportion is potentially fermentable in colon. In contrast to cereals, beans are rich in the raffinose family oligosaccharides (RFO, galacto-oligosaccharides (GOS)); mainly raffinose, stachyose, and verbascose, resulting in 3.2 g RFO (mainly raffinose) in the evening meal with brown beans, compared with minute amounts of RFO in the WWB portion. In pigs [25] and in in vitro models of colonic fermentation [20], [25], it has been shown that GOS increase the formation of propionate and butyrate.

It is possible that differences between e.g. barley kernels and brown beans in botanical integrity and composition of colonic substrates may affect the metabolism of the gut microbiota differently, thus explaining some of the differences seen in host metabolic responses. Both beans and cereals contain other bio active components which also may infer metabolic effects. However, the present study with beans confirm previous data with barley kernels suggesting benefits of evening meals rich in indigestible carbohydrates on key metabolic parameters in an over-night perspective.

In this study, a brown bean evening meal yielded substantial effects on satiety- and hunger hormones the next morning. Consequently, plasma PYY was increased by 51%, and ghrelin lowered by 14%, in comparison with the WWB evening meal. The concentrations of PYY in the morning after brown beans were inversely correlated to a decreased willingness to eat. The over-night effects on appetite sensations are an important finding, which may be useful in the dietary control and maintenance of a healthy weight. Previously, 2 weeks dietary supplementation with inulin (16 g/day) in healthy normal weighted subjects [26] resulted in lowered hunger rates, increased satiety hormones (GLP-1 and PYY) and improved glucose tolerance. In addition, feeding oligofructose to obese mice for 14 weeks increased gut bifidobacteria [27]. The increase in bifidobacteria was accompanied by improved glucose regulation, a reduced inflammatory tonus, and increased breath H_2_ excretion, indicating a prebiotic mechanism. The metabolic benefits obtained in mice fed with diets supplemented with pure fructooligosaccharides thus appear similar to those obtained in the current over-night study in healthy humans, indicating a prebiotic effect also of the intrinsic indigestible carbohydrates present in boiled brown beans. Consequently, it is possible that prebiotic benefits on metabolic risk variables may occur in an over-night perspective e.g. already within 11–14 hours. To our knowledge, this is the first time prebiotic over-night effects of beans are reported in healthy subjects. The indigestible substrates that potentially may be responsible for the metabolic benefits in the present study include fermentable poly- and oligosaccharides, including RS and RFO. Also polyphenols, which are present in beans [28] may constitute a colonic substrate [29].

In rats, GOS stimulated growth of bifidobacteria after 4 and 7 days, and promoted maintenance of the intestinal barrier function in rats with impaired barrier function [30]. The gut hormone GLP-2 has implication in gut barrier functions by reducing the intestine wall permeability to e.g. endotoxins. It has been shown in mice models that prebiotic (oligofructose) modulation of the gut microbiota improves intestinal barrier functions by mechanisms involving GLP-2; resulting in reduced endotoxaemia and systemic inflammation [31]. Low-grade chronic systemic inflammation is associated with obesity and insulin resistance [32]. A diet induced increase in endotoxemia and increased low grade inflammation has been demonstrated in mice models when fed with a high fat diet for 4 weeks [33]. The increase in endotoxemia was accompanied by an increased proportion of an LPS-containing microbiota [33]. Interestingly, the present study revealed an increase in p-GLP-2 at breakfast after an evening meal with brown beans, and a concomitant decrease in plasma of inflammatory markers (IL-6 and IL-18). The relationships between gut microflora and lowered inflammation previously seen in mice makes it feasible to suggest that the decreased concentrations of inflammatory markers obtained in the present study in part may be related to an improved gut barrier, involving increased GLP-2.

The gut peptides GLP-1, GLP-2 and oxyntomoduline are proglucagon derived peptides, secreted from intestinal L-cells in the small intestine and colon. Although not proglucagon derived, also PYY are secreted from L-cells in the gut. The gut peptides play an important role in energy homeostasis, e.g. glucose- and appetite regulation [34], [35]. GLP-1 and PYY are considered anti-diabetic and anti-obesity hormones, and GLP-1-based therapies are currently used as a novel treatment for type 2 diabetes [36]. The present and previous [9] studies show that release of gut peptides are affected by choice of diet, and increased by certain indigestible colonic substrates. Dietary stimulation of endogenous gut peptide secretion thus may be a promising approach for prevention and treatment of metabolic diseases. All together the present study shows that, within a time frame of 11–14 h, brown beans are potent in this respect. No effects of brown beans were however detected on GLP-1 concentrations in the current study, despite the significant effects obtained on other gut peptides also released, or even co-secreted, from L-cells [37].

BCFA are involved in the regulation of intestinal Na absorption, and contribute to some extent to intestinal energy supply [23], [38]. However, the role of BCFA produced by the gut microbiota in relation to systemic metabolism is poorly elucidated. On the contrary, several metabolic effects have been suggested for SCFA. In rats fed RS for 10 days, GLP-1 and PYY secretion were stimulated, and it was shown that bacterial fermentation and production of SCFA in the gut were associated with increased proglucagon and PYY gene expression [39]. SCFA are produced during colonic fermentation of indigestible carbohydrates, and in addition to providing energy to colonic enterocytes, SCFA also function as signaling molecules. Consequently, it has been demonstrated that SCFA produced by bacterial fermentation may trigger signalling cascades through acting on SCFA receptors (FFAR2 and FFAR3) on L-cells (in vitro model), resulting in increased release of gut peptides such as GLP-1 and PYY [40]. The increase in PYY concentrations seen in the present study can thus be suggested to result from the increased production of SCFA. SCFA receptors are not only found in the intestine, but are expressed also in e.g. immune cells and human white adipose tissues [41]. Obesity, insulin resistance and type 2 diabetes are closely associated with chronic inflammation, predominately in adipose tissue [42]. In adipose tissue, propionate has been found to be a ligand for the SCFA-SCFA receptors [41], and in an adipose tissue cell culture, propionate was shown to have anti-inflammatory effects [43]. The effects were accompanied by improved expression of lipoprotein lipase and GLUT4. These effects on adipocytes in cell culture provide a link between colonic fermentation- and anti-diabetic effects, and are coherent with the presently reported results in humans.

The present study had certain limitations. An evident constraint is the unbalanced gender participation since 10 out of the 16 participants were women. However, an ANOVA calculations of glucose IAUC (primary outcome) revealed that there were no main effects of gender, or gender*treatment interactions (*p*>0.05).

The study design was an “over-night” study to investigate effects of the test product in an 11–14 h perspective. The design was set in part to overcome the inconvenience it may have caused the participants of staying at the experimental department over a whole day (from morning to evening). The over-night metabolic effects seen on the test variables can probably partly be generalised to concern similar metabolic effects if the test product was consumed in the morning, and test variables determined in a similar time perspective over the day.

In summary, it was shown that an evening meal of boiled brown beans, in comparison with WWB, have beneficial effects on glucose regulation, satiety, and inflammatory markers at a following standardized breakfast. Plasma propionate and isobutyrate were significantly increased after brown beans compared to after WWB. The study indicates that colonic fermentation of intrinsic indigestible carbohydrates present in certain foods, e.g. in brown beans, may constitute a mechanism for a promising approach aiming at dietary prevention and/or treatment of obesity and the metabolic syndrome.

## Supporting Information

Protocol S1 **Trial protocol.**
(DOCX)

Checklist S1 **CONSORT checklist.**
(DOC)

## References

[1] Feldeisen SE, Tucker KL (2007) Nutritional strategies in the prevention and treatment of metabolic syndrome. Appl Physiol Nutr Metab. 32: 46–60. 10.1139/h06-101 17332784

[2] Tovar J, Nilsson A, Johansson M, Ekesbo R, Aberg AM, et al (2012) A diet based on multiple functional concepts improves cardiometabolic risk parameters in healthy subjects. Nutrition & metabolism. 9: 29. 10.1186/1743-7075-9-29 PMC3361470 22472183

[3] Jenkins DJ, Kendall CW, Augustin LS, Franceschi S, Hamidi M, et al (2002) Glycemic index: overview of implications in health and disease. Am J Clin Nutr. 76: 266S–73S. 10.1093/ajcn/76/1.266S 12081850

[4] McKeown NM, Meigs JB, Liu S, Wilson PW, Jacques PF (2002) Whole-grain intake is favorably associated with metabolic risk factors for type 2 diabetes and cardiovascular disease in the Framingham Offspring Study. Am J Clin Nutr. 76: 390–8. 10.1093/ajcn/76.2.390 12145012

[5] Micallef MA, Garg ML (2009) Anti-inflammatory and cardioprotective effects of n-3 polyunsaturated fatty acids and plant sterols in hyperlipidemic individuals. Atherosclerosis. 204: 476–82. 10.1016/j.atherosclerosis.2008.09.020 18977480

[6] Thorburn A, Muir J, Proietto J (1993) Carbohydrate fermentation decreases hepatic glucose output in healthy subjects. Metabolism. 42: 780–5. 10.1016/0026-0495(93)90249-n 8510524

[7] Nilsson A, Östman E, Preston T, Bjorck I (2008) Effects of GI vs content of cereal fibre of the evening meal on glucose tolerance at a subsequent standardized breakfast. EJCN. 62: 712–20. 10.1038/sj.ejcn.1602784 17522615

[8] Nilsson A, Granfeldt Y, Östman E, Preston T, Bjorck I (2006) Effects of GI and content of indigestible carbohydrates of cereal-based evening meals on glucose tolerance at a subsequent standardised breakfast. EJCN. 60: 1092–9. 10.1038/sj.ejcn.1602423 16523203

[9] Nilsson AC, Östman EM, Holst JJ, Bjorck IM (2008) Including indigestible carbohydrates in the evening meal of healthy subjects improves glucose tolerance, lowers inflammatory markers, and increases satiety after a subsequent standardized breakfast. J Nutr. 138: 732–9. 10.1093/jn/138.4.732 18356328

[10] Nilsson AC, Östman EM, Knudsen KE, Holst JJ, Bjorck IM (2010) A cereal-based evening meal rich in indigestible carbohydrates increases plasma butyrate the next morning. J Nutr. 140: 1932–6. 10.3945/jn.110.123604 20810606

[11] Papanikolaou Y, Fulgoni VL, 3rd (2008) Bean consumption is associated with greater nutrient intake, reduced systolic blood pressure, lower body weight, and a smaller waist circumference in adults: results from the National Health and Nutrition Examination Survey 1999–2002. J Am Coll Nutr. 27: 569–76. 10.1080/07315724.2008.10719740 18845707

[12] Sichieri R (2002) Dietary patterns and their associations with obesity in the Brazilian city of Rio de Janeiro. Obes Res. 10: 42–8. 10.1038/oby.2002.6 11786600

[13] Sievenpiper JL, Kendall CW, Esfahani A, Wong JM, Carleton AJ, et al (2009) Effect of non-oil-seed pulses on glycaemic control: a systematic review and meta-analysis of randomised controlled experimental trials in people with and without diabetes. Diabetologia. 52: 1479–95. 10.1007/s00125-009-1395-7 19526214

[14] Wong CL, Mollard RC, Zafar TA, Luhovyy BL, Anderson GH (2009) Food intake and satiety following a serving of pulses in young men: effect of processing, recipe, and pulse variety. J Am Coll Nutr. 28: 543–52. 10.1080/07315724.2009.10719786 20439550

[15] Tovar JG, Y Björck (1992) I (1992) Effect of Processing on Blood Glucose and Insulin Responses to Starch in Legumes. J. Agric. Food Chem. 40: 1846–1851.

[16] Brighenti F (1998) Summary of the conclusion of the working group on Profibre interlaboratory study on determination of short chain fatty acids in blood. In: Functional Properties of Non-digestible Carbohydrates. F. Gullion. R. Amadò, M. T. Amaral-Collaco, H. Andersson, N. G. Asp, K. E. Bach Knudsen, M. Champ, J. Mathers, J. A. Robertson, I. Rowland, and J. Van Loo, ed. European Commission, DG XII, Science, Research and Development, Brussels, Belgium. 150–153.

[17] Björck IME, Siljeström M (1992) In-Vivo and In-Vitro Digestibility of Starch in Autoclaved Pea and Potato Products. J Sci Food Agric. 58: 541–553.

[18] Åkerberg AKE, Liljeberg HGM, Granfeldt YE, Drews A, Björck IM (1998) An in vitro method, based on chewing, to predict resistant starch content in foods allows parallel determination of potentially available starch and dietary fibre. J Nutr. 128: 651–659. 10.1093/jn/128.3.651 9482777

[19] Asp N-G, Johansson C-G, Hallmer H, Siljeström M (1983) Rapid enzymatic assay of insoluble and soluble dietary fibre. J Agric Food Chem. 31: 476–482. 10.1021/jf00117a003 6309935

[20] Hernandez-Salazar M, Osorio-Diaz P, Loarca-Pina G, Reynoso-Camacho R, Tovar J, et al (2010) In vitro fermentability and antioxidant capacity of the indigestible fraction of cooked black beans (Phaseolus vulgaris L.), lentils (Lens culinaris L.) and chickpeas (Cicer arietinum L.). J Sci Food Agric. 90: 1417–22. 10.1002/jsfa.3954 20549791

[21] Henningsson AM, Nyman EM, Bjorck IM (2001) Content of short-chain fatty acids in the hindgut of rats fed processed bean (Phaseolus vulgaris) flours varying in distribution and content of indigestible carbohydrates. Br J Nutr. 86: 379–89. 10.1079/bjn2001423 11570990

[22] Mortensen PB, Clausen MR, Bonnen H, Hove H, Holtug K (1992) Colonic fermentation of ispaghula, wheat bran, glucose, and albumin to short-chain fatty acids and ammonia evaluated in vitro in 50 subjects. JPEN. Journal of parenteral and enteral nutrition. 16: 433–9. 10.1177/0148607192016005433 1331553

[23] Nyangale EP, Mottram DS, Gibson GR (2012) Gut microbial activity, implications for health and disease: the potential role of metabolite analysis. J Proteome Res. 11: 5573–85. 10.1021/pr300637d 23116228

[24] Jie Z, Bang-Yao L, Ming-Jie X, Hai-Wei L, Zu-Kang Z, et al (2000) Studies on the effects of polydextrose intake on physiologic functions in Chinese people. Am J Clin Nutr. 72: 1503–9. 10.1093/ajcn/72.6.1503 11101478

[25] Smiricky-Tjardes MR, Grieshop CM, Flickinger EA, Bauer LL, Fahey GC Jr (2003) Dietary galactooligosaccharides affect ileal and total-tract nutrient digestibility, ileal and fecal bacterial concentrations, and ileal fermentative characteristics of growing pigs. Journal of animal science. 81: 2535–45. 10.2527/2003.81102535x 14552381

[26] Cani PD, Lecourt E, Dewulf EM, Sohet FM, Pachikian BD, et al (2009) Gut microbiota fermentation of prebiotics increases satietogenic and incretin gut peptide production with consequences for appetite sensation and glucose response after a meal. Am J Clin Nutr. 90: 1236–43. 10.3945/ajcn.2009.28095 19776140

[27] Cani PD, Neyrinck AM, Fava F, Knauf C, Burcelin RG, et al (2007) Selective increases of bifidobacteria in gut microflora improve high-fat-diet-induced diabetes in mice through a mechanism associated with endotoxaemia. Diabetologia. 50: 2374–83. 10.1007/s00125-007-0791-0 17823788

[28] Xu BJ, Yuan SH, Chang SK (2007) Comparative analyses of phenolic composition, antioxidant capacity, and color of cool season legumes and other selected food legumes. J Food Sci. 72: S167–77. 10.1111/j.1750-3841.2006.00261.x 17995859

[29] Parkar SG, Stevenson DE, Skinner MA (2008) The potential influence of fruit polyphenols on colonic microflora and human gut health. Int J Food Microbiol. 124: 295–8. 10.1016/j.ijfoodmicro.2008.03.017 18456359

[30] Zhong Y, Cai D, Cai W, Geng S, Chen L, et al (2009) Protective effect of galactooligosaccharide-supplemented enteral nutrition on intestinal barrier function in rats with severe acute pancreatitis. Clin Nutr. 28: 575–80. 10.1016/j.clnu.2009.04.026 19525042

[31] Cani PD, Possemiers S, Van de Wiele T, Guiot Y, Everard A, et al (2009) Changes in gut microbiota control inflammation in obese mice through a mechanism involving GLP-2-driven improvement of gut permeability. Gut. 58: 1091–103. 10.1136/gut.2008.165886 PMC2702831 19240062

[32] Wellen KE, Hotamisligil GS (2005) Inflammation, stress, and diabetes. J Clin Invest. 115: 1111–9. 10.1172/JCI25102 PMC1087185 15864338

[33] Cani PD, Amar J, Iglesias MA, Poggi M, Knauf C, et al (2007) Metabolic endotoxemia initiates obesity and insulin resistance. Diabetes. 56: 1761–72. 10.2337/db06-1491 17456850

[34] Stanley S, Wynne K, Bloom S (2004) Gastrointestinal satiety signals III. Glucagon-like peptide 1, oxyntomodulin, peptide YY, and pancreatic polypeptide. Am J Physiol Gastrointest Liver Physiol. 286: G693–7. 10.1152/ajpgi.00536.2003 15068960

[35] Dalvi PS, Belsham DD (2012) Glucagon-like peptide-2 directly regulates hypothalamic neurons expressing neuropeptides linked to appetite control in vivo and in vitro. Endocrinology. 153: 2385–97. 10.1210/en.2011-2089 22416082

[36] Ahren B (2007) GLP-1-based therapy of type 2 diabetes: GLP-1 mimetics and DPP-IV inhibitors. Curr Diab Rep. 7: 340–7. 10.1007/s11892-007-0056-9 18173966

[37] Baggio LL, Huang Q, Brown TJ, Drucker DJ (2004) Oxyntomodulin and glucagon-like peptide-1 differentially regulate murine food intake and energy expenditure. Gastroenterology. 127: 546–58. 10.1053/j.gastro.2004.04.063 15300587

[38] Musch MW, Bookstein C, Xie Y, Sellin JH, Chang EB (2001) SCFA increase intestinal Na absorption by induction of NHE3 in rat colon and human intestinal C2/bbe cells. Am J Physiol Gastrointest Liver Physiol. 280: G687–93. 10.1152/ajpgi.2001.280.4.G687 11254495

[39] Zhou J, Martin RJ, Tulley RT, Raggio AM, McCutcheon KL, et al (2008) Dietary resistant starch upregulates total GLP-1 and PYY in a sustained day-long manner through fermentation in rodents. Am J Physiol Endocrinol Metab. 295: E1160–6. 10.1152/ajpendo.90637.2008 PMC2584810 18796545

[40] Tolhurst G, Heffron H, Lam YS, Parker HE, Habib AM, et al (2012) Short-chain fatty acids stimulate glucagon-like peptide-1 secretion via the G-protein-coupled receptor FFAR2. Diabetes. 61: 364–71. 10.2337/db11-1019 PMC3266401 22190648

[41] Al-Lahham SH, Roelofsen H, Priebe M, Weening D, Dijkstra M, et al (2010) Regulation of adipokine production in human adipose tissue by propionic acid. Eur J Clin Invest. 40: 401–7. 10.1111/j.1365-2362.2010.02278.x 20353437

[42] Hotamisligil GS (2006) Inflammation and metabolic disorders. Nature. 444: 860–7. 10.1038/nature05485 17167474

[43] Al-Lahham S, Roelofsen H, Rezaee F, Weening D, Hoek A, et al (2012) Propionic acid affects immune status and metabolism in adipose tissue from overweight subjects. Eur J Clin Invest. 42: 357–64. 10.1111/j.1365-2362.2011.02590.x 21913915

[44] Åkerberg AKE, Liljeberg H, Bjorck I (1998) Effects of amylose/amylopectin ratio and baking conditions on resistant starch formation and glycaemic indices. journal of cereal science. 28: 71–80.

[45] Howlett JF, Betteridge VA, Champ M, Craig SA, Meheust A, et al.. (2010) The definition of dietary fiber - discussions at the Ninth Vahouny Fiber Symposium: building scientific agreement. Food & nutrition research. 54. 10.3402/fnr.v54i0.5750 PMC2972185 21052531

