# Supplementary material for: Effects of a Brown Beans Evening Meal on Metabolic Risk Markers and Appetite Regulating Hormones at a Subsequent Standardized Breakfast: A Randomized Cross-Over Study
Source: PLoS One. 2013 Apr 5;8(4):e59985. doi: 10.1371/journal.pone.0059985 (PMC3618511; doi:10.1371/journal.pone.0059985)
Supplement: Protocol S1 — Trial protocol. (DOCX) [file pone.0059985.s001.docx]

**Protocol S1. Trial protocol.**

**An English translation (Google translation with minor necessary changes) of the Swedish trial protocol.**

**“Effects on glucose tolerance and appetite at breakfast following an evening meal with test products”**

**Research Plan**
**Background**Large and frequent fluctuations in blood sugar levels is an important risk factor for cardiovascular disease. For people at risk, glucose tolerance deteriorates gradually over a longer period of time without fasting glucose values ​​change. Studies have shown that postprandial glycemia may be a stronger predictor of cardiovascular disease than fasting glucose, and therefore increases interest steadily in various ways to reduce the blood glucose response after a meal. One of the mechanisms underlying risks of high blood sugar levels is that they contribute to increased inflammatory activity in the body, which itself is thought to play a major role in atherosclerosis process. In fact, type 2 diabetics have a chronic higher degree of systemic inflammation, and it has proved possible to obtain a regression in atherosclerosis progression by improving glycemic control.
Observational studies have recently provided many ideas for interesting risk markers that give a picture of the inflammatory process, and examples of these are: IL-6, TNF-α, CRP and adiponectin. IL-6 is a marker for insulin resistance and a predictor of the development of type 2 diabetes. Both diabetic and obese have elevated levels of IL-6. Adiponectin is a substance with anti atherosclerosis properties, and it has in animal models been found that supplementation with adiponectin increases insulin sensitivity.
With the above as a starting point, it is very important to make efforts to bring down the blood glucose response after a meal. We have long had a focus on the acute processes, but later there has been an increase in understanding of how glucose metabolism is affected in the longer term. In so-called "second meal" studies, we examine how glucose tolerance after a standardized meal may be affected by a previous meal. The majority of low-GI foods may improve glycemic control at a subsequent meal served 4-5 hours after the first, while low-GI foods also contain a high amount of indigestible carbohydrates may affect glucose tolerance at a meal served after 10 - 11 hours. It turns out to be a clear link between colon metabolism and glucose regulation, primarily through the short-chain fatty acids formed during the fermentation of indigestible substrates, but also through the release of GLP-1; a insulin stimulating gut hormone. An example of food systems that have interesting implications in this context is cooked barley. We have seen an improvement in both glucose, insulin, free fatty acids, IL-6 and adiponectin at a standardized breakfast preceded by a late evening meal with cooked barley kernels. When the corresponding cereals instead were served as a breakfast, glucose tolerance was improved not only at a second-meal lunch, but also at a subsequent third meal (dinner). In addition to glucose regulation, also appetite regulation is an important area. One factors which have proved interesting in connection to appetite regulation is the incretin GLP-1. GLP-1 has shown to reduce the gastric emptying rate (so called ileal brake). When barley kernels were served as a late evening meal, we have seen increased GLP-1 levels at the following breakfast. In addition to GLP-1, we evaluate also hunger peptides (ghrelin), with the hope of establishing a relationship between ghrelin and other metabolic parameters, and possibly with the subjective rating of hunger, satiety, willingness to eat, or voluntary energy intake. New knowledge about the parameters that control energy intake is valuable to be able to design foods that can not only improve glucose control but also can contribute to sustainable weight control.

**Objective**The aim of the current study is to evaluate how different combinations of resistant starch and dietary fiber affect glucose tolerance and other risk markers for cardiovascular disease, perceived saturation and hydrogen excretion (marker of colon fermentation) in an "over-night" perspective. By determine blood parameters and hydrogen excretion the day after the test subjects have consumed test products for evening meal, important insights can be obtained regarding which time frame different types of resistant starch and / or fiber causes fermentation peaks that may affect glucose tolerance and other risk markers. The long-term goal is to tailor foods that contribute to good blood sugar control and appetite sensations at a specific time interval after intake.

**Study design**
The test subjects consume either test or reference product the the day prior to the visit at the research department. They also asked not to expose themselves to strenuous physical activity or drinking alcohol during the day prior to the visit. On the morning of trial, the research subjects arrive fasting. An intravenous canula is inserted, and capillary and venous blood samples are withdrawn before the standardized breakfast meal (0 min) and then at 15, 30, 45, 60, 90 and 120 min after breakfast. In those cases metabolic parameters are followed also after a following standardized lunch, the meal is served 3.5 to 4.5 hours after breakfast. Immediately before lunch, and after 15, 30, 45, 60, 90 and 120 min venous and capillary blood samples are withdrawn. The subjects can spend the time in- between tests freely, however, they are not allowed to perform physical activity or food intake. In each test series, six to eight blood tests are withdrawn capillary and venouse for each test subject. The test days are separated by at minimum one week, and normally a test series is ongoing during three to four months.

**Analyses and blood volumes**The total blood volume during a trial day is at maximum 75 ml. Cumulative amount of blood in the implementation of eight test meals is 600 ml, spread over 3-4 months. The capillary blood samples were taken to measure blood glucose, while the venous blood is used for determining insulin, glucagon, incretins (GIP, GLP-1), free fatty acids, triglycerides, short chain fatty acids, IL-6, IL-18, adiponectin and ghrelin. All parameters are not measured at every test points. The reason for not determine glood glucose from venous blood is that it has been shown that capillary blood is a more sensitive and accurate measurements of postprandial glucose concentrations. Hydrogen determination is made using a hydrogen analyzer which subjects exhale into every half hour throughout the trial day. Perceived satiety / hunger is assessed on a visual scale at the same time points as hydrogen measurements.

**Description of test products and meals**Test products as research subjects will eat for supper consists of cooked grains or bread with different fiber, resistant starch and sometimes cooked cereal grains. White bread is used as reference product. All test products are approved. The maximum of eight different test and reference products in each test series will be eaten in random order. The breakfast will be standardized in terms of quality and there will be sandwiches with or without butter and toppings. In some series the breakfast is standardized in quantity to study glucose tolerances, while in other cases ad libitum intake is studied where subjects are told to eat to a moderate level of saturation. In the latter case the food intake are documented. The standardized lunch consists either of fried meatballs and mashed potatoes or hash-in-pan. Water is served with all meals and coffee / tea or water is served at one point during the trial days.

**Test subjects**

Test subjects (20 people per study) consists mainly of students, graduate students, or employees at Lund University, and they are not in a position of dependence to the investigator. Some of the subjects have previously participated in similar studies, however we always make sure that the same person does not participate in more than two studies per year with each study requires 500 ml of blood or more. Recruitings are made through local bulletin boards, e-mail or telephone. Additionally, test subjects disseminate to others about our research and need for subjects, which results in people become interested and get in touch. Research subjects must be healthy, normal weight (BMI 19-25) and aged 20 to 35 years.

**Risks and possible incidents**Risks connecting to blood sampling are considered very low. There are no risks of incidents linked to experimental products because test subjects are asked about food allergies and / or intolerances before being allowed to participate in a study.

**Registration of the experimental data**All data will be recorded on paper in connection with blood sampling, data in anonymous form used in computer programs for statistical analysis and results will only be reported on the static representation group level.

**Forskningsplan**

**Bakgrund**

Stora och frekventa svängningar i blodsockernivåer är en viktig riskfaktor för hjärt-kärlsjukdom. För människor i riskzonen kan glukostoleransen försämras gradvis under en längre tid utan att fasteglukosvärdena förändras. Studier har visat att postprandiell glykemi kan vara en starkare prediktor för hjärt-kärlsjukdom än fasteglukos och därför ökar intresset stadigt för att på olika sätt reducera blodsockersvaren efter måltid. En av mekanismerna bakom riskerna med för höga blodsockernivåer är att de bidrar till en ökad inflammatorisk aktivitet i kroppen, som i sig tros spela stor roll för åderförkalkningsprocessen. Faktum är att typ 2 diabetiker har en konstant ökad grad av systemisk inflammation och det har visat sig möjligt att få en tillbakagång i atherosklerosutvecklingen hos dessa genom en bättre blodsockerkontroll.
Observationsstudier har på senare tid gett många uppslag till intressanta riskmarkörer som ger en bild av inflammationsprocessen och exempel på dessa är: IL-6, TNF-α, CRP och adiponectin. IL-6 är en markör för insulinresistens och en prediktor för utveckling av typ 2 diabetes. Både diabetiker och feta har förhöjda nivåer av bl.a. IL-6. Adiponectin är en substans med anti-atherogena egenskaper och man har i djurmodeller sett att supplementering med adiponectin ökar insulinkänsligheten.

Med ovanstående som utgångspunkt är det fortsatt mycket intressant och viktigt att göra insatser för att få ned blodsockersvaren efter måltid. Vi har under lång tid haft fokus på de akuta förloppen men efterhand ökar också våra kunskaper om hur glukosmetabolismen påverkas i ett längre perspektiv. I så kallade ”second meal”-studier undersöker vi hur glukostoleransen efter en standardiserad måltid påverkas av vad man ätit måltiden innan. Merparten av låg-GI-livsmedlen kan förbättra blodsockerregleringen vid en efterföljande måltid som serveras 4-5 timmar efter den första, medan de låg-GI-livsmedel som även innehåller en hög mängd odigererbara kolhydrater kan påverka glukostoleransen vid en måltid som serveras efter 10-11 timmar. Det visar sig finnas en tydlig koppling mellan colon-metabolism och glukosreglering och då främst via de kortkedjiga fettsyror som bildas vid fermenteringen av odigererbart material, men också via frisättning av GLP-1 som är ett insulinstimulerande tarmhormon. Ett exempel på livsmedelssystem som har intressanta effekter i detta sammanhang är kokta kornkärnor. Vi har sett en förbättring av både glukos, insulin, fria fettsyror, IL-6 och adiponectin vid en standardiserad frukost som föregåtts av ett sent kvällsmål med kokta kornkärnor. När motsvarande kornkärnor istället serverades som en frukost förbättrades glukostoleransen inte bara vid den efterföljande lunchen utan även vid ett tredje standardiserat mål på kvällen. Förutom glukosregleringen i sig är också aptitreglering ett viktigt område. En av de faktorer som visat sig intressanta i mättnadssammanhang är GLP-1, som bl.a. har en hämmande effekt på magsäckens tömningshastighet (sk. ileal brake) och i fallet då kornkärnor serverats som kvällsmål har vi sett ökade GLP-1 nivåer vid den påföljande frukosten. Förutom GLP-1 utvärderar vi också hungerpeptiden ghrelin, med hopp om att kunna fastställa ett samband mellan ghrelin och någon annan metabol parameter samt eventuellt den subjektiva hunger-/mättnadsbedömningen och/eller frivilligt energiintag. Nya kunskaper om vilka parametrar som styr energiintaget är värdefulla för att kunna designa livsmedel som inte bara kan förbättra glukosregleringen utan också kan bidra till en hållbar viktreglering.

**Målsättning**

Målsättningen med de aktuella studierna är att utvärdera hur olika kombinationer av resistent stärkelse och kostfiber påverkar glukostolerans och andra riskmarkörer för hjärt-kärlsjukdom, upplevd mättnad och vätgasutsöndring (colonfermentering) i ett ”över-natten” perspektiv. Genom att följa blodparametrar och vätgasutsöndring under dagen efter det att forskningspersonerna ätit en testprodukt till kvällsmat erhålls viktiga kunskaper om när olika typer av resistent stärkelse och/eller fiber ger upphov till fermenteringstoppar som kan påverka glukostolerans eller andra riskmarkörer. Den långsiktiga målsättningen är att kunna skräddarsy livsmedel som bidrar till en god blodsockerreglering och en bra mättnadskänsla vid en given tidpunkt efter intaget.

**Studiernas upplägg**

Forskningspersonen äter dagen innan besöket ett kvällsmål bestående av antingen test- eller referensprodukt. De uppmanas dessutom att inte utsätta sig för hård fysisk aktivitet eller dricka alkohol under dagen före besöket. På försöksdagens morgon kommer forskningspersonerna fastande till försöksavdelningen. En venflon sätts in i forsknings-personens armveck och kapillära och venösa blodprover tas före den standardiserade frukostmåltiden (0 min) och sedan vid 15, 30, 45, 60, 90 och 120 min efter frukosten. I de fall även nästa mål studeras ges en standardiserad lunch 3,5-4,5 h efter frukosten. Omedelbart före lunchen samt efter 15, 30, 45, 60, 90 och 120 min tas venösa och kapillära blodprover. Tiden mellan provtagningarna disponeras fritt av forskningspersonerna, dock tillåts ej fysisk aktivitet eller intag av föda. I varje testserie kommer varje forkningsperson att inta sex till åtta test- och referensmåltider med minst en veckas mellanrum, normalt pågår en testserie i tre till fyra månader.

**Analyser och blodmängder**

Den sammanlagda blodvolymen under en försöksdag uppgår till max 75 ml. Ackumulerad blodmängd vid genomförandet av åtta testmåltider blir 600 ml, fördelat över 3-4 månader. De kapillära blodproverna tas för att mäta blodsocker medan det venösa blodet används för bestämning av insulin, glukagon, inkretiner (GIP, GLP-1), fria fettsyror, triglycerider, kortkedjiga fettsyror, IL-6, IL-18, adiponektin och ghrelin. Alla parametrar mäts inte vid varje tidpunkt. Anledningen till att proven för analys av blodsocker ej tas i venkatetern är att det har visats att kapillärt tagna blodsocker är mer känsliga och rättvisande vid postprandiella mätningar. Vätgasbestämning görs med hjälp av en vätgasanalysator som försökspersonerna får blåsa i en gång i halvtimmen under hela försöksdagen. Upplevd mättnad/hunger bedöms av forskningspersonerna på en visuell skala i samband med vätgasmätningarna.

**Beskrivning av testprodukter och måltider**

Testprodukterna som forskningspersonerna kommer att äta till kvällsmat består av kokta spannmålskärnor alternativt bröd med olika innehåll av kostfiber, resistent stärkelse och i vissa fall kokta spannmålskärnor. Vitt bröd används som referensprodukt. Alla testprodukter utgörs av godkända livsmedel och livsmedelstillsatser. De maximalt åtta olika test- och referensprodukterna i respektive testserie kommer att ätas i slumpmässig ordning.

Frukostmålet kommer att standardiseras avseende kvalitet och det blir smörgåsar med eller utan smör och pålägg. I någon testserie standardiseras ävan kvantiteten av frukostmålet för att studera glukotolerans medan i andra fall studeras ett ad libitum-intag till en lagom nivå av mättnad. I det sistnämnda fallet kommer matintaget att dokumenteras. Det standardiserade lunchmålet består antingen av stekta köttbullar och potatismos eller pytt-i-panna. Vatten serveras till alla måltiderna och kaffe/te eller vatten serveras vid ett tillfälle under försöksdagen.

**Forskningspersoner**

Forskningspersonerna (20 personer per studie) utgörs i huvudsak av studenter, doktorander eller anställda vid Lunds Universitet och de är inte i beroendeställning till försöksledaren. Några av forskningspersonerna har tidigare deltagit i liknande studier, vi ser dock alltid till att samma person inte deltar i mer än två studier per år där respektive studie kräver 500 ml blod eller mer. Kontakter sker genom lokala anslagstavlor, e-post eller telefon. Dessutom sprider forskningspersonerna själva information om vårt behov av forskningspersoner vilket leder till att intresserade tar kontakt. Forskningspersonerna skall vara friska, normalviktiga (BMI 19-25) och i åldern 20 till 35 år.

**Risker och möjliga tillbud**

Eventuella risker med blodprovstagning bedöms som mycket ringa. Ingen risk för tillbud kopplat till försöksprodukterna finns då försökspersonerna tillfrågas om livsmedelsallergi och/eller intoleranser innan de tillåts delta i en studie.

**Registrering av försöksdata**

All data kommer att registreras på papper i anslutning till provtagningen, data kommer i avidentifierad form användas i dataprogram för statistisk bearbetning och resultat kommer endast att redovisas på statisktisk gruppnivå.
