# Supplementary material for: Effects of a Brown Beans Evening Meal on Metabolic Risk Markers and Appetite Regulating Hormones at a Subsequent Standardized Breakfast: A Randomized Cross-Over Study
Source: PLoS One. 2013 Apr 5;8(4):e59985. doi: 10.1371/journal.pone.0059985 (PMC3618511; doi:10.1371/journal.pone.0059985)
Supplement: Checklist S1 — CONSORT checklist. (DOC) [file pone.0059985.s002.doc]

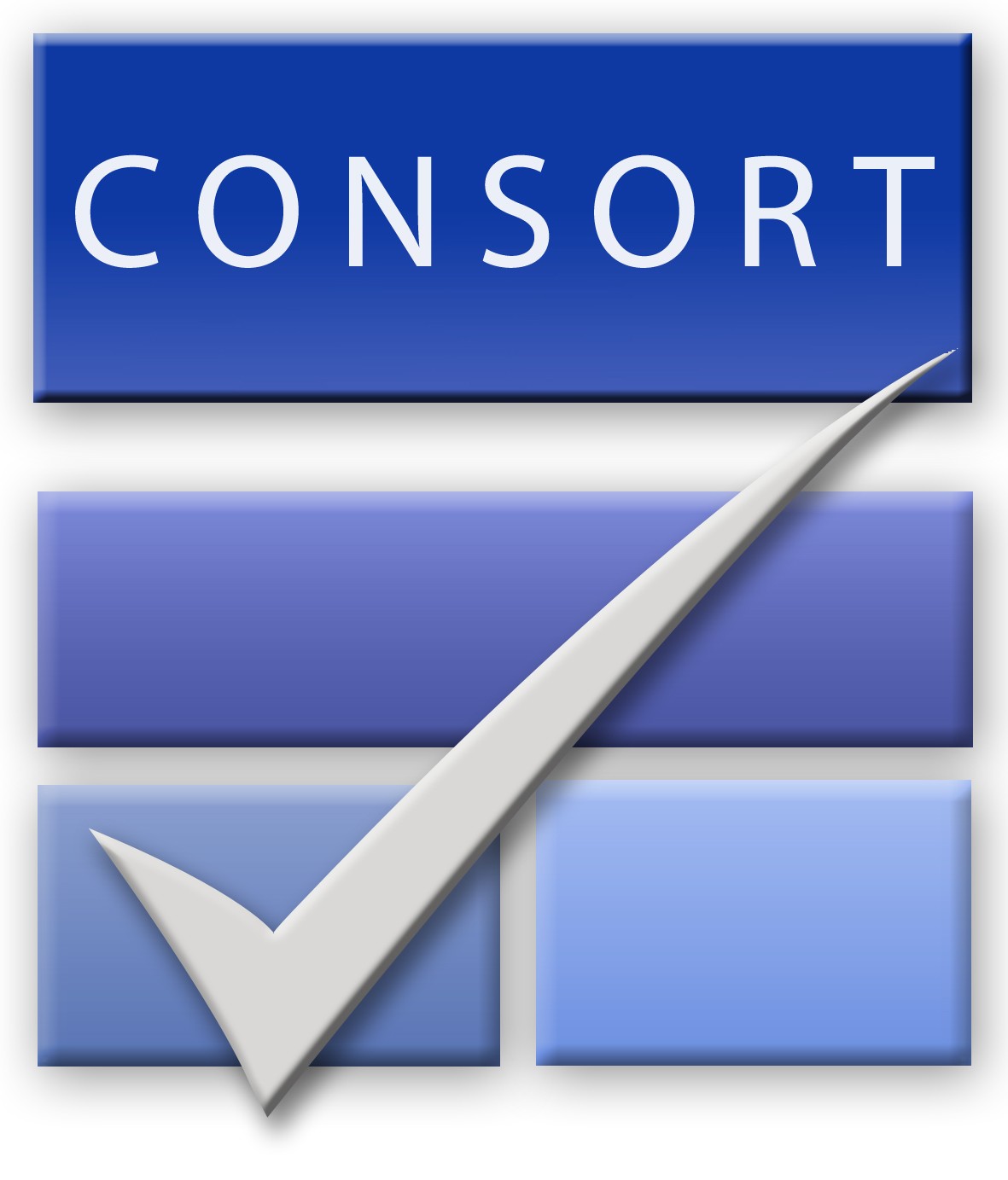
Checklist S1. CONSORT 2010 checklist of information to include when reporting a randomised trial*

| Section/Topic | Item No | Checklist item | | Reported on page No |
| --- | --- | --- | --- | --- |
| Title and abstract | | | | |
|  | 1a | Identification as a randomised trial in the title | | - |
|  | 1b | Structured summary of trial design, methods, results, and conclusions (for specific guidance see CONSORT for abstracts) | | abstract |
| Introduction | | | | |
| Background and objectives | 2a | Scientific background and explanation of rationale | | Introduction |
|  | 2b | Specific objectives or hypotheses | | Introduction |
| Methods | | | | |
| Trial design | 3a | Description of trial design (such as parallel, factorial) including allocation ratio | | Material and methods (Experimental procedure) |
|  | 3b | Important changes to methods after trial commencement (such as eligibility criteria), with reasons | | - |
| Participants | 4a | Eligibility criteria for participants | | Material and methods (Test subjects) |
|  | 4b | Settings and locations where the data were collected | | Material and methods (Experimental procedure) |
| Interventions | 5 | The interventions for each group with sufficient details to allow replication, including how and when they were actually administered | | Material and methods (Test subjects, The evening meal test product and the WWB reference bread, Standardised breakfast, Physiological test variables, Chemical analyses of the test- and reference products, Experimental procedure, Calculations and statistical methods) |
| Outcomes | 6a | Completely defined pre-specified primary and secondary outcome measures, including how and when they were assessed | | Material and methods (Physiological test variables, Chemical analyses of the test- and reference products, Experimental procedure, Calculations and statistical methods) |
|  | 6b | Any changes to trial outcomes after the trial commenced, with reasons | | - |
| Sample size | 7a | How sample size was determined | | Material and methods (Power calculations) |
|  | 7b | When applicable, explanation of any interim analyses and stopping guidelines | | - |
| Randomisation: |  |  | |  |
| Sequence generation | 8a | Method used to generate the random allocation sequence | | Material and methods (Experimental procedure) |
|  | 8b | Type of randomisation; details of any restriction (such as blocking and block size) | | - |
| Allocation concealment mechanism | 9 | Mechanism used to implement the random allocation sequence (such as sequentially numbered containers), describing any steps taken to conceal the sequence until interventions were assigned | | - |
| Implementation | 10 | Who generated the random allocation sequence, who enrolled participants, and who assigned participants to interventions | | The test leader AN randomise the order of test products, and LE enrolled the participants and assigned the interventions. |
| Blinding | 11a | If done, who was blinded after assignment to interventions (for example, participants, care providers, those assessing outcomes) and how | | - |
|  | 11b |  | | - |
| Statistical methods | 12a | Statistical methods used to compare groups for primary and secondary outcomes | | Material and methods (Calculations and statistical methods) |
|  | 12b | Methods for additional analyses, such as subgroup analyses and adjusted analyses | | - |
| Results | | | | |
| Participant flow (a diagram is strongly recommended) | 13a | For each group, the numbers of participants who were randomly assigned, received intended treatment, and were analysed for the primary outcome | | Material and methods (Test subjects, Experimental procedure, Calculations and statistical methods, CONSORT flow diagram figure 1 ) |
|  | 13b | For each group, losses and exclusions after randomisation, together with reasons | | Material and methods (CONSORT flow diagram figure 1) |
| Recruitment | 14a | Dates defining the periods of recruitment and follow-up | | Material and methods (Test subjects) |
|  | 14b | Why the trial ended or was stopped | | - |
| Baseline data | 15 | A table showing baseline demographic and clinical characteristics for each group | | Material and methods (Test subjects) and Results.  The study included one test product and one reference product. No base line data was obtained; the results after the reference product are taken as base line values. |
| Numbers analysed | 16 | For each group, number of participants (denominator) included in each analysis and whether the analysis was by original assigned groups | | All participants (sixteen) were included in all analyses. |
| Outcomes and estimation | 17a | For each primary and secondary outcome, results for each group, and the estimated effect size and its precision (such as 95% confidence interval) | | Results |
|  | 17b | For binary outcomes, presentation of both absolute and relative effect sizes is recommended | | - |
| Ancillary analyses | 18 | Results of any other analyses performed, including subgroup analyses and adjusted analyses, distinguishing pre-specified from exploratory | | - |
| Harms | 19 | All important harms or unintended effects in each group (for specific guidance see CONSORT for harms) | | No side effects were detected or reported. |
| Discussion | | | | |
| Limitations | 20 | Trial limitations, addressing sources of potential bias, imprecision, and, if relevant, multiplicity of analyses | Discussion | |
| Generalisability | 21 | Generalisability (external validity, applicability) of the trial findings | Discussion | |
| Interpretation | 22 | Interpretation consistent with results, balancing benefits and harms, and considering other relevant evidence | Discussion | |
| Other information | | |  | |
| Registration | 23 | Registration number and name of trial registry | ClinicalTrials.gov NCT01706042. | |
| Protocol | 24 | Where the full trial protocol can be accessed, if available | Protocol S1 (Trial protocol) | |
| Funding | 25 | Sources of funding and other support (such as supply of drugs), role of funders | This study was funded by the Lund University Antidiabetic Food Center, a VINNOVA VINN Excellence Center | |

*We strongly recommend reading this statement in conjunction with the CONSORT 2010 Explanation and Elaboration for important clarifications on all the items. If relevant, we also recommend reading CONSORT extensions for cluster randomised trials, non-inferiority and equivalence trials, non-pharmacological treatments, herbal interventions, and pragmatic trials. Additional extensions are forthcoming: for those and for up to date references relevant to this checklist, see [www.consort-statement.org](http://www.consort-statement.org/).
